# Adherence to outpatient cardiac rehabilitation and related factors in STEMI after PCI in China: a sequential explanatory mixed method study protocol

**DOI:** 10.3389/fcvm.2025.1542942

**Published:** 2025-08-05

**Authors:** Wenjie Zhang, Hongjin Wang, Peiru Li, Beibei Wu, Xiaodong Wang

**Affiliations:** ^1^ Health Management Center, Affiliated Hospital of Jiangsu University, Zhenjiang, China; ^2^ Department of Respiratory Medicine, Affiliated Hospital of Jiangsu University, Zhenjiang, China; ^3^ Cardiovascular Department, Affiliated Hospital of Jiangsu University, Zhenjiang, China

**Keywords:** adherence, cardiac rehabilitation, health belief model, acute myocardial infarction, percutaneous coronary intervention, mixed-methods study

## Abstract

**Background:**

Acute myocardial infarction remains a major contributor to global morbidity and mortality. Cardiac rehabilitation is widely recognized as an essential component of the comprehensive medical management for patients with ST-elevation myocardial infarction, especially among those who have undergone percutaneous coronary intervention. Adherence to cardiac rehabilitation encompasses patient collaboration with a healthcare provider, active involvement in the treatment regimen, and persistence in practice, characterized by self-efficacy and relapse prevention. Outpatient cardiac rehabilitation constitutes the second phase of the continuum of care, bridging the inpatient and long-term maintenance stages. However, adherence to outpatient cardiac rehabilitation programs in China remains suboptimal. The factors influencing outpatient cardiac rehabilitation in patients with ST-elevation myocardial infarction after percutaneous coronary intervention have not yet been fully elucidated.

**Aims:**

To investigate the status and influencing factors of adherence to outpatient cardiac rehabilitation in patients with ST-elevation myocardial infarction after percutaneous coronary intervention one month after discharge in China and gain a deeper understanding of this phenomenon of interest.

**Methods:**

An explanatory sequential mixed-methods design will be employed to conduct this study with three phases. The first phase is a quantitative study with a cross-sectional design to assess the level of adherence to outpatient cardiac rehabilitation and related influences based on the Health Belief Model among 198 patients with ST-elevation myocardial infarction after percutaneous coronary intervention in two tertiary hospitals in Zhenjiang, Jiangsu Province, China. The second phase is followed by a qualitative study to explore the patients’ perceived facilitators and barriers to adherence to outpatient cardiac rehabilitation. Purposive sampling, semi-structured interviews, and conventional content analysis approaches will be used to collect and analyze the data. The final phase links to integrating the data and developing a targeted strategy to improve adherence to outpatient cardiac rehabilitation among patients with ST-elevation myocardial infarction after percutaneous coronary intervention. The nominal group technique and &quot;weaving techniques&quot; will be implemented in accordance with the results of the preceding two phases.

**Discussion:**

A targeted strategy to improve outpatient cardiac rehabilitation adherence would be designed considering the factors affecting adherence to outpatient cardiac rehabilitation in patients with ST-elevation myocardial infarction after percutaneous coronary intervention.

**Clinical Trial Registration:**

This study has been registered in the Chinese Clinical Trial Registry: ChiCTR2400080035.

## 1 Background

Acute myocardial infarction (AMI) is a leading cause of global morbidity and mortality. In recent years, the incidence and mortality of AMI have steadily increased annually in most countries (1, 2). Traditionally, AMI has been classified into ST-elevation MI (STEMI) and non-ST-elevation MI (NSTEMI) (3). Survivors of AMI in Asian countries have relatively high mortality rates. In China, the mortality rates for hospitalization, 30-day, and 90-day were 4.0%, 5.9%, and 7.6%, respectively (4). AMI also entails a significant financial and social burden, such as missed work, prescription expenses, and hospital stay (5). Percutaneous coronary intervention (PCI) is the preferred treatment for AMI revascularization (6). An increase of 1% in PCI cases was associated with a 0.72% decrease in mortality rate (7). According to data from the Chinese National Center for Cardiovascular Quality Improvement, 86.8% of STEMI patients in tertiary hospitals had reperfusion, with emergency PCI accounting for 98.37% of cases (8). Nonetheless, the hospital mortality rate of patients with STEMI after PCI (STEMI-PCI) reached 5.3% in Shanghai (9, 10) and 26.08% in Hebei (11), and the 30-day mortality rate in Sichuan was 12.7% (12).

The American Heart Association (AHA) underscored the significance of cardiac rehabilitation (CR) in patients with AMI and demanded prompt attention to this worldwide issue (13). CR can be divided into three phases: inpatient, outpatient, and maintenance phase (13). In hospitalized and outpatient patients with recent myocardial infarction (MI), PCI, CABG, stable angina, and similar conditions, the AHA placed a strong emphasis on CR (14). The World Health Organization (WHO) defined “adherence” as “the extent to which a person's behavior – taking medication, following a diet, and/or executing lifestyle changes, corresponds with agreed recommendations from a health care provider” in 2003 (15). After that, scholars proposed a conceptual analysis and delineated “adherence to CR” as “patients' collaboration with a healthcare provider, active involvement in the treatment regimen, and persistence in practice characterized by self-efficacy and relapse-prevention” (16).

Numerous benefits have been empirically supported and thoroughly demonstrated (13, 17, 18). These benefits include cardiac risk prevention (19), reduction of cardiac and all-cause mortality rates by approximately 30% and 15% at 1-year follow-up (14), and improvement of quality of life (QoL) and negative emotions (14, 20). However, hospital-based CR participation rates among eligible outpatient patients globally remain at a low level of 10%–30%, due to obstacles such as low socio-economic position, accessibility, conflicting commitments, and cost (21). As a result, home-based CR has gained popularity in the UK, Canada, and Australia, and the adherence rate of STEMI-PCI patients to OCR has remained between 22% and 80% (22, 23). However, this situation is not optimistic in China. The adherence rate has only reached 7.05%–29.9% (24, 25), although the rate among inpatients was 72.74% (26). Chinese scholars have released a summary of multiple pieces of evidence related to CR (27, 28) and have suggested that patients in stable conditions can more flexibly switch to home-based CR from hospital- or center-based CR to improve adherence with appropriate self-monitoring methods.

Despite the general recognition of the benefits of CR adherence, dropout rates exceed 80% (29, 30). Numerous studies have shown a significant increase in the risk of death and MI when CR programs are not adhered to (13), and the influencing factors are multifactorial and individualistic (31). According to an Australian study, the factors included male sex, employment status, health support, shorter hospital stays, and previous AMI or PCI (23). However, in China, adherence was adversely correlated with age, male sex, hyperlipidemia, and D2B time, but positively connected to educational level and arrhythmia during PCI (25). Additional research has noted that adherence to OCR is statistically influenced by patient recognition (32), depression (33, 34), and self-efficacy (35, 36). Diabetes, smoking, and CR costs (22, 37) were all linked to poor OCR adherence. A qualitative study found that STEMI-PCI patients may not be able to recognize and manage ischemic symptoms immediately, may have psychological problems during the acute and recovery stages (38), as well as often overestimate their level of physical activity after discharged (39). More importantly, few studies have investigated effective strategies for improving the level of adherence to OCR in STEMI-PCI patients. The identification of both facilitators and obstacles to adherence to OCR and the mechanisms involved have not been evaluated.

The Health Belief Model (HBM) (40) is a theoretical framework designed to measure the motivations behind starting and continuing health behaviors. It considers demographic variables, psychological characteristics, patients' beliefs about their health, barriers and beneﬁts for certain health behaviors, self-efficacy, and actions triggered by cues (41). HBM has been widely applied in behavioral prevention (42). Even so, no study has found how STEMI-PCI patients' health beliefs actively initiate health behaviors, how their views matter, or how they adhere to OCR. In addition, there are notable differences in the efficacy of influencing factors in different contexts (e.g., gender and social support) (43). Considering the significance and variances in culture, the economy, and society, an inclusive approach is required to address this issue. A mixed-methods design enables researchers to use a qualitative lens to explain the findings of quantitative results by exploring informants' perceptions in greater detail and, thus, a deeper understanding of the phenomenon than either method alone (44). Therefore, it is important and urgent to determine the level of adherence to OCR in STEMI-PCI patients and related influencing factors based on HBM. This will also serve as a basis for early identification and precise control to improve adherence to OCR, particularly in China.

### 1.1 Study aim

This study aims to identify the level and factors influencing adherence to home-based OCR in STEMI-PCI patients within one month after discharge and related influencing factors (quantitative phase), then explore the perceived facilitators and barriers of adherence to OCR (qualitative phase), and finally, gain a deeper understanding to develop target strategies to improve adherence to CR in China.

### 1.2 Specific objectives

1. Determining the level and factors influencing adherence to OCR among STEMI-PCI patients one month after discharge in two tertiary hospitals in Zhenjiang, Jiangsu Province, China.
2. Exploring perceived facilitators and obstacles of adherence to OCR among STEMI-PCI patients one month after discharge from two tertiary hospitals in Zhenjiang, Jiangsu Province, China.
3. Generating a deeper understanding of adherence to OCR in STEMI-PCI patients and developing targeted strategies to improve adherence to OCR.

### 1.3 Definition of terms for this study

Adherence to CR is the behavior of AMI-PCI patients who are actively involved in the home-based CR program after discharge. It will be measured using the Chinese version of the Adherence to Cardiac Rehabilitation Scale (C-ACRS), developed by Wen Xiaohui (45), which includes five dimensions: physician-prescribed exercise, medication management, nutrition management, cardiac risk factor modification, and psychological management.

## 2 Methods/design

### 2.1 Study design

**Figure 1:**
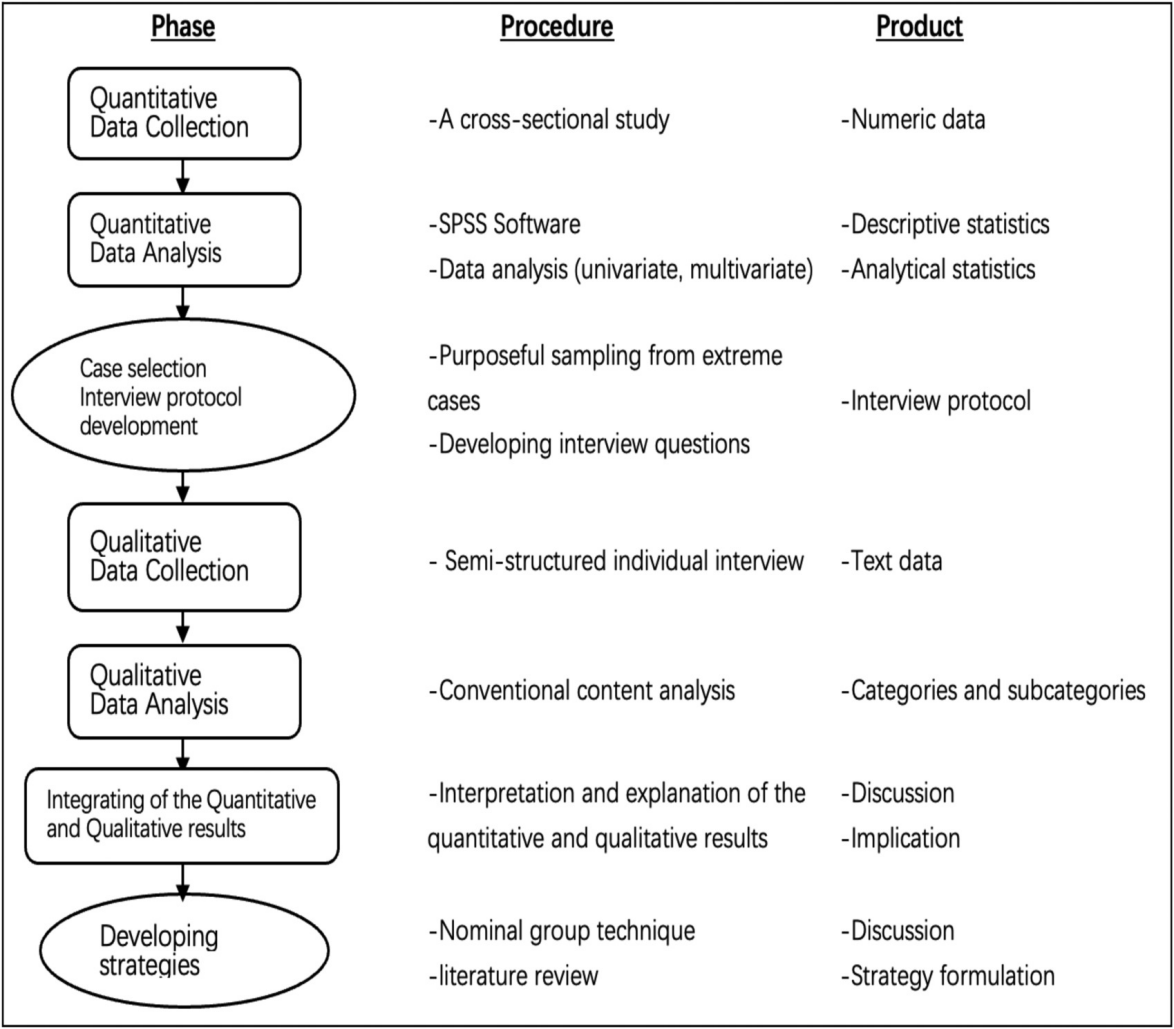
Study diagram of the sequential explanatory mixed-method approach.

This study uses a sequential explanatory mixed-method approach for data collection, analysis, and integration of the quantitative and qualitative phases (Figure 1). Based on post-positivism, the mixed-method paradigm combines quantitative and qualitative methods to improve our understanding of OCR adherence in patients undergoing STEMI-PCI. Qualitative data will be expanded to quantitative data in the first phase, and both quantitative and qualitative data will be integrated subsequently. These findings will contribute to targeted strategies for improving adherence to OCR.

### 2.2 Phase one: quantitative study

Phase one is designed as a cross-sectional study that will be carried out to assess the level of adherence to OCR of STEMI-PCI patients one month after discharge from two tertiary hospitals in Zhenjiang, Jiangsu Province, China. Then, the impact of demographic variables, psychological characteristics, health beliefs, and adherence to OCR, as well as the related factors influencing adherence, will be investigated.

#### 2.2.1 Sample size and sampling method

According to the suggestion of Nunnally and Bernstien, selecting 5–10 participants per item is appropriate for multivariate analysis (46) 165 participants will be recruited to unitize the factor analysis for the 33 items of the Scale of Adherence to Cardiac Rehabilitation scale (ACRS). Considering a follow-up attrition rate of 20%, this number will increase to 198.

Following the approval of the research project by the Ethics Committee of the two hospitals, the study will be conducted on 198 participants with AMI after PCI one month after discharge who receive home-based OCR. The researchers will visit two hospitals, identify eligible patients using the hospital information system (HIS) following the proportion (70% and 30%) of the target population in two hospitals, explain the study objectives and brief process to them, and obtain informed consent to participate. Patients will complete the Demographic Data Sheet (DDS).

#### 2.2.2 Inclusion criteria

Patients who (1) are aged ≥18 years; (2) are diagnosed with AMI (I21of ICD-10 standard); (3) have received PCI; (4) participate in the home-based phase 2 CR; (5) can speak, read, and understand Mandarin; and (6) are willing to participate in this survey.

#### 2.2.3 Exclusion criteria

Patients who (1) have complications from PCI and (2) participate in the phase 2 CR program in the hospital as outpatients will be excluded.

#### 2.2.4 Research instrument

##### 2.2.4.1 The demographic data sheet (DDS)

The researcher will develop a DDS, which will include two parts: demographic data, such as age, sex, marital status, educational level, employment status, and smoking status, and history of illness, such as diabetes, dyslipidemia, length of stay, D2B time, and arrhythmia during PCI. The DDS will be a form of open-ended and closed-ended questions.

##### 2.2.4.2 The center for epidemiological studies depression scale (CES-D-S)

Feng et al. (47) developed a Chinese version of the CES-D-S to measure depression. This scale contains four dimensions with 10 items: physical factors, interpersonal relationships, negative emotions, and positive emotions. The I-CVIs ranged from 0.830–1.000, while the S-CVI was 0.86. CFA showed that the structural validity model achieved adaption (RMSEA <0.08, GFI and CFI >0.90, and CMIN/df <3) (47). Each item is rated on a 4 Likert scale from 0 to 3, with a total score range of 0–30 points. The higher the score, the more severe the depression.

##### 2.2.4.3 The multidimensional scale of perceived social support (MSPSS)

The MSPSS evaluates perceived social support and was translated into Chinese by Chen et al. (48). The Chinese Version of the MSPSS has 12 items with three dimensions: family support, friend support, and other important support. The CFA of this scale supported a three-factor first-order model that fit well (RMSEA = 0.070, CFI = 0.941, and *χ*^2^/*df* = 2.753) (48). Each item is rated on a 7 Likert-type scale, where 1 means “strongly disagree,” and 7 means “strongly agree.” The total score ranges from 12 to 84 points. Higher scores represent higher perceived social support.

##### 2.2.4.4 The coronary artery disease health belief scale (HBMS)

The HBMS was developed by Zhang et al. (49) in Chinese based on the HBM to assess patients' health beliefs related to behavior change. It contains five dimensions: perceived susceptibility, perceived seriousness, perceived benefit, perceived barriers, and cues to action. with 27 items. Eight common factors were extracted from the EFA of this scale, and the cumulative variance contribution rate was 60.698%. The correlation between the subscales and the total scale ranged from 0.811 to 0.876 (49). Each item was rated on a 5 Likert–type scale ranging from 1 (strongly disagree) to 5 (strongly agree). The total score ranged from 27 to 135. Higher scores indicate higher health beliefs.

##### 2.2.4.5 The Chinese version of the cardiac self-efficacy scale (C-CSES)

The C-CSES was developed by Zhang et al. (50) and was translated from the CSEQ developed by Sullivan et al. (51) includes 13 items with three dimensions: control illness, control symptoms, and maintain function. The I-CVIs of this scale ranged from 0.81–0.96, while the S-CVI was 0.87. Confirmatory factor analysis (CFA) supported a three-factor high-order structure of the C-CSES with model fit indices: RMSEA = 0.084, CFI = 0.954, NNFI = 0.927, IFI = 0.954 and *χ*^2^/*df* = 2.572 (50). Each item was rated on a 5 Likert–type scale ranging from 0 (not at all) to 4 (completely confident). The total score ranged from 0 to 52 points. Higher scores indicate higher self-efficacy.

##### 2.2.4.6 The scale of adherence to cardiac rehabilitation (ACRS)

The ACRS was developed by Wen, X.H. (52) based on quality interviews and literature reviews to assess adherence to CR in CHD patients. The ACRS contains 33 items with five subscales: exercise, medication, risk factors, nutritional, and psychological management. The I-CVIs of the scale ranged from 0.80–1.00, while the S-CVI was 0.96. The cumulative variance contribution rate was 60.698%, and the CFA showed good structural validity: *X*^2^ < 0.001, RMSEA = 0.055, CFI = 0.851 (52). The scale responds “yes” or “no” for each item (yes = 1 point, no = 0 points). The total score range of the scale is 0–33 points. The total score is classified into high and low: 33–25 points indicates high adherence to cardiac rehabilitation and 24–0 indicates low adherence to cardiac rehabilitation (45).

#### 2.2.5 Data collection

This study will collect quantitative data using questionnaires. The following stages will be implemented to complete the data collection process.

(1) After obtaining permission from the directors of each hospital, the researcher will ask one research coordinator from the cardiac clinic of each hospital and a quiet, private room at the cardiac clinic to collect the form and questionnaires.
(2) The researcher will prepare 200 packages, each containing a form and five questionnaires, and explain the data collection process to the research coordinators.
(3) The researcher will obtain a list of STEMI PCI patients for follow-up at the outpatient department. The researcher will screen eligible patients and assign numerical codes to replace the patients' names. Subsequently, the researcher will provide a coded list to the research coordinators for subject selection.
(4) The research coordinator will schedule an appointment in advance for potential patients at the cardiac clinic after they meet the doctor and explain the study's purposes, procedures, duration, and benefits. If the subjects agree to participate, they will be requested to sign a consent form.
(5) The research coordinator distributes the research package to all participants. Each participant will spend 45–60 min completing the questionnaires and will then return the questionnaires and consent form separately in two designated boxes within the room. The research coordinator then checks for the completeness of all documents collected from the boxes and returns them to the researcher every week.

#### 2.2.6 Data analysis

IBM SPSS 22.0 software will be used for data analysis. Statistical significance will be set at *p* < 0.05. The data analysis process was as follows:

The independent variables, including demographic variables, psychological characteristics, and the six constructs of the HBM, are described using frequency, percentage, mean, and standard deviation.

Dummy coding will be performed for categorical data. The assumption of logistic regression, including linearity, homoscedasticity, and residual normality, will be checked before entering the data into SPSS. Linearity: The partial regression plot between dependent and independent variables should be random. Homoscedasticity: Check the residual plot of the dependent variable; the scatter plot should be random without a curve pattern. Normality of residuals: Check the histogram of the dependent variable and a standard probability plot. Univariate analysis and binary logistic multivariate statistical analysis will be used to calculate the regression coefficient, odds ratio (OR), and 95% confidence interval of each variable using the stepwise entry method, and the independent risk factors related to adherence to home-based phase 2 CR will be analyzed.

### 2.3 Phase two: qualitative study

This phase consists of an exploratory qualitative study with conventional content analysis. Using this approach, perceived facilitators and obstacles to adherence to OCR will be investigated and explained.

#### 2.3.1 Sampling method

Purposive sampling will be employed to explore patients' perceived facilitators and barriers to adherence to OCR within one month after discharge. Qualitative interviews were conducted after completion of the quantitative phase. Extreme cases on both sides of the overall ACRS score spectrum from phase one (the uppermost and lowermost 10% extreme scores of different subgroups of adherence) will be selected as participants in this phase.

#### 2.3.2 Data collection

(1) Gaining access: The researcher will explain the purpose of the study to the directors of the selected hospitals to gain administrative permission and support.
(2) Recruiting informants: The researcher will communicate and invite potential informants to participate in the interview. Informants who are willing to participate and available for the time will be interviewed by the researcher. Usually, 20–30 patients of them are invited to participate in interviews. Purposive sampling will be used to recruit informants, some from the high-adherence group and others from the low-adherence group. The interview guide will design questions based on the quantitative study findings before the qualitative phase. The time will be set based on the agreement between the patients and the researcher.
(3) Data collection: Digital audio recorders were used during the interviews after obtaining verbal permission. The time for the interview will be 60–90 min.
(4) Establishing rapport and trust:
  (a) The research coordinator will receive informants with greetings and help them be seated in a quiet private room for the interview in the cardiac clinic and introduce herself to the patients. The research objectives and research questions will be introduced, and patients will be asked to sign an informed consent form.
  (b) Project posters will be placed to ensure that all informants understand the purposes and procedures.
  (c) General questions of the informants will be asked before to increase their comfort and level of confidence among them. Then, the interview questions will be gradually raised according to the interview guidelines.

#### 2.3.3 Data analysis

Qualitative data will be analyzed using a conventional content analysis approach, which is typically employed with a study design whose goal is to characterize a phenomenon when there is little current theory or research literature on a topic (53). Using this approach, data analysis begins by reading all the data repeatedly to achieve immersion and obtain a sense of the whole. The data were then read word by word to derive codes by first highlighting the exact words from the text to capture critical thoughts or concepts. Next, the researchers make notes of their first impressions, thoughts, and initial analysis. Following this, code labels appear, which are typically the original coding scheme and are frequently taken straight from the text. The researcher selects preliminary codes after open-coding three to four transcripts, which can then be applied to the remaining transcripts. When data do not fit into an existing code, new codes may be added. Consequently, the codes are grouped into categories that make sense. Based on their distinctions and commonalities, these codes are divided into main categories and subcategories. To interpret the underlying meaning, this method makes it possible to extract both manifest content and implicit context (54). The accuracy of the qualitative data will be assessed in this study using traditional criteria (Credibility, Dependability, Confirmability, Transferability) (55). NVivo software (version 12.0) will be employed to manage the interview data.

The “following a thread” method will be used to analyze the data. It begins by analyzing the data of each component to locate important conclusions and unanswered questions. Researchers refer to this as a thread when they take a category or theme from one component and follow it throughout others (56). “Extreme case analysis” is an additional method that involves identifying extreme cases by continuously analyzing datasets. High- and low-extreme cases will be used to demonstrate a substantial association between patients' health perceptions and adherence to OCR.

### 2.4 Phase three: integration and development

Conventional content analysis generally yields an interpretation that offers a comprehensive response to research questions while staying more in line with the phenomenon. Researchers must combine content categories to create a relevant panorama and cogent narrative to accomplish this in practice. It is critical to convey the intricacy of the facts within the larger narrative or interpretation. The key conclusions of both quantitative and qualitative data are presented side by side and narratively reported through “weaving techniques” in data integration. Together, these two sets of data can yield new insights and additional information through joint displays, which offer a deeper and more comprehensive explanation of participant responses and research problems (44).

The nominal group technique (NGT) will be used to develop appropriate strategies to promote adherence to OCR in patients undergoing STEMI-PCI. NGT is a structured group-based technique used to establish a consensus (57). Here's an overview:

• Expert session: We will convene a panel of cardiologists, CR specialists, general practitioners, cardiac nurses, nutritionists, and relevant stakeholders.
• Strategy presentation: We will introduce a set of proposed effective strategies derived from earlier research phases.
• Input collection: Participants and their families will be invited to provide feedback aimed at refining and prioritizing these strategies.
• Synthesis and refinement: The strategies will be further refined based on expert input to ensure they are evidence-based and effective.

## 3 Discussion

The main points of lowering the 30-day mortality rate of AMI-PCI patients after discharge and raising patient quality of life are adhering to CR. The causes of non-adherence or low adherence rates, however, are many and complex.

The level and indicators pertaining to adherence to OCR in STEMI-PCI patients should be determined to achieve this goal. This is the first study to examine the level and relevant factors impacting adherence to OCR in STEMI-PCI patents and to design targeted improvement strategies by exploring adherence to OCR using an explanatory sequential mixed-method. This study's design advances our knowledge of the variables that affect OCR adherence by supporting the integration of various viewpoints, strategies, and techniques in line with the principles of epistemological pluralism. To help STEMI-PCI patients achieve better health outcomes, the strategy suggested in this study may help increase awareness among CR providers and policymakers, particularly those pertaining to medical insurance.
